# Self-Efficacy and Perceived Study-Related Difficulties in Early Medical Training: A Correlational Study

**DOI:** 10.1007/s40670-026-02895-1

**Published:** 2026-09-26

**Authors:** Juliana Silva Arruda, Letícia Bernardine Silva Arruda, Isaac Coelho Mitoso, Hermano Alexandre Lima Rocha, Marcos Kubrusly, Felipe Queiroz Siqueira

**Affiliations:** 1 University Christus, R. João Adolfo Gurgel, 133 - Cocó, Fortaleza, CE 60190-180 Brazil; 2 Community Health Department, Federal University of Ceará, R. Papi Júnior, 1223 - Rodolfo Teófilo, Fortaleza, CE 60430-235 Brazil

**Keywords:** Self-efficacy, Medical education, Perceived study-related difficulties, Academic performance, Self-regulated learning

## Abstract

Self-efficacy is a relevant psychosocial variable in medical education because it may influence how students organize their learning, persist when facing academic demands, and cope with perceived difficulties. This study analyzed the relationship between perceived self-efficacy and perceived study-related difficulties among medical students in the first cycle of training, corresponding to the early stages of medical education with an emphasis on basic sciences and foundational knowledge.A correlational cross-sectional study was conducted with 118 medical students enrolled between the first and fourth semesters at a Brazilian higher education institution. Participants completed an adapted version of the Self-Efficacy in Higher Education Scale and a questionnaire addressing difficulties during study, including academic performance, content mastery, examination preparation, opportunities to clarify doubts, and time management. Descriptive statistics and Pearson bivariate correlations were calculated using Jamovi software. Bonferroni correction was applied to reduce type I error. The mean score for study difficulties was 2.13±0.74. Self-efficacy dimensions showed high mean scores, including academic self-efficacy *(*8.08 ± 1.34), self-efficacy in training regulation (8.40 ± 1.26), proactive self-efficacy (8.09 ± 1.39), social-interaction self-efficacy (8.39 ± 1.33), and academic-management self-efficacy (8.59 ± 1.31). Study difficulties were negatively correlated with all self-efficacy dimensions, especially academic self-efficacy (*r* = − .530; IC 95% [-0.650; − 0.385]; R² = 0.281 ) and self-efficacy in training regulation (*r* = − .414; IC 95% [− 0.553; −0.252], R² = 0.171). These findings highlight the importance of early institutional interventions focused on self-regulated learning and academic support in medical education.

**Supplementary Information:**

The online version contains supplementary material available at https://doi.org/10.1007/s40670-026-02895-1.

## Introduction

Medical education is commonly associated with high academic demands, intense workloads, and the need for sustained self-directed learning. Students entering medical school often encounter a transition that requires not only cognitive engagement but also emotional regulation, planning, persistence, and adaptation to new pedagogical models. These challenges may be particularly relevant during the first semesters, when students are still developing strategies to manage academic pressure, understand complex biomedical content, prepare for assessments, and balance study routines with personal life.

In this context, self-efficacy has been identified as a central construct for understanding how students interpret their abilities and respond to academic demands. Bandura defined self-efficacy as the belief in one’s capability to organize and execute actions required to attain desired outcomes [1, p. 3]. Self-efficacy does not merely reflect objective skill; rather, it influences the choices individuals make, the effort they invest, their persistence when obstacles arise, and how they interpret emotional and physiological states during challenging situations. In higher education, such beliefs may affect learning behaviors, academic engagement, and students’ willingness to seek support when difficulties emerge.

Self-efficacy is especially relevant in medical education because students are expected to progressively assume greater responsibility for their own learning. Active methodologies, problem-based learning, and competency-based curricula require learners to identify knowledge gaps, plan study goals, monitor progress, and use feedback constructively [2]. Students who believe they are capable of learning autonomously may be more likely to engage in self-regulated learning strategies, such as goal setting, strategic planning, self-monitoring, and reflective evaluation. Zimmerman’s model of self-regulated learning emphasizes self-efficacy as a key motivational factor in the cyclical process through which learners plan, perform, monitor, and adapt their academic behavior [3].

From a multidimensional perspective, different domains of self-efficacy may be related to perceived study-related difficulties through distinct aspects of students’ academic experience [3]. Academic self-efficacy concerns students’ perceived capability to learn, understand, and demonstrate academic content and may therefore be more directly related to difficulties involving performance and content mastery. Self-efficacy in training regulation reflects confidence in planning, monitoring, and adjusting one’s learning process, making it particularly relevant to challenges involving study organization, examination preparation, and time management. Proactive self-efficacy may influence students’ willingness to seek resources and take initiative when difficulties arise, whereas social-interaction self-efficacy may facilitate help-seeking, communication with peers and faculty, and opportunities to clarify doubts. Academic-management self-efficacy, in turn, concerns students’ perceived ability to navigate academic demands and institutional routines. Thus, although these domains are related, they represent different aspects of students’ perceived capabilities and may not be equally associated with the difficulties experienced during medical training.

Previous studies have suggested that self-efficacy is associated with empathy, attitudes toward mental health, academic performance, and psychological adjustment among medical students [4–6]. Research on self-regulated learning also indicates that effective study strategies, such as practice testing, spaced practice, and self-explanation, tend to require adequate levels of motivation and perceived control over learning [7]. Thus, self-efficacy may play an important role during the transition into medical school by helping students persist, organize their learning, and reinterpret difficulties as manageable challenges rather than evidence of incapacity.

Despite growing evidence linking self-efficacy to academic performance, psychological adjustment, and self-regulated learning, less is known about how distinct dimensions of self-efficacy are associated with specific perceived study-related difficulties during the early stages of medical training. Previous research has often examined self-efficacy in relation to broader academic or psychological outcomes, providing less insight into its relationship with everyday learning challenges such as content mastery, examination preparation, time management, and opportunities to clarify doubts [2, 5].

In this study, perceived study-related difficulties are defined as students’ self-reported challenges in dealing with core academic demands during medical training, including performance requirements, examination preparation, closing knowledge gaps, and mastering the volume of learning content.

This distinction is particularly relevant during the first semesters of medical school, when students are adapting to new workloads, pedagogical approaches, and expectations for autonomous learning. By examining multiple domains of self-efficacy alongside specific perceived study-related difficulties among first-cycle medical students, the present study provides a more differentiated understanding of the relationship between students’ efficacy beliefs and their perceived study-related difficulties. In Brazilian medical education, the first cycle—commonly referred to as the basic cycle—corresponds to the initial phase of undergraduate medical training, predominantly focused on basic sciences and foundational knowledge and competencies for subsequent clinical training. Therefore, this study aimed to analyze the relationship between perceived self-efficacy and the frequency of perceived study-related difficulties among first-cycle medical students. Specifically, it sought to examine the strength of correlations between these difficulties and different domains of self-efficacy.

## Materials and Methods

### Study Design

This was a cross-sectional correlational study linked to a broader research project on learning strategies and mental health among medical students. The study was designed to examine associations between perceived self-efficacy and difficulties experienced during study activities.

### Participants and Setting

Participants were 118 medical students enrolled between the first and fourth semesters at Universidade Christus (Unichristus), Fortaleza, Ceará, Brazil. The sample was selected by convenience and included students who voluntarily agreed to participate and completed the online instruments anonymously. Participants had a mean age of 22.87 years, ranging from 17 to 52 years. Most participants identified as female (72.3%), followed by male (26.9%); a small proportion preferred not to report gender (0.8%).

### Instruments

Self-efficacy was assessed using an adapted version of the Self-Efficacy in Higher Education Scale for medical students [8]. The scale included the following dimensions: academic self-efficacy, self-efficacy in training regulation, proactive self-efficacy, social-interaction self-efficacy, and academic-management self-efficacy. Evidence of construct validity for the original five-factor structure was previously established through factor analysis. This instrument demonstrated high overall internal consistency (Cronbach’s α = 0.94), with coefficients ranging from 0.80 to 0.88 across the five dimensions. Perceived study-related difficulties were assessed using a questionnaire addressing difficulties during study, including perceived problems related to academic performance, content mastery, preparation for examinations, opportunities to clarify doubts, and time management [9]. The questionnaire demonstrated high overall internal consistency (Cronbach’s α = 0.81). An open-ended sociodemographic question about perceived study-related difficulties was also included in the original data collection instrument, although the present analysis focused on quantitative indicators.

The two constructs were analyzed differently because their original instruments have distinct conceptual and scoring structures. Self-efficacy was examined through five separate subscales because the Self-Efficacy in Higher Education Scale was developed and validated as a multidimensional measure, with five theoretically and psychometrically established domains [8]. In contrast, the Questionnaire on Difficulties During Studies was designed to capture an overall perception of difficulties experienced during study through the related items [9]. Following the scoring procedure described in the original study, these items were combined into a single mean composite score. Thus, the different analytical approaches reflected the theoretical and scoring structures of the respective instruments rather than an arbitrary decision made for the present analysis.

### Data Collection

Data were collected between March and April 2025 using online forms distributed to eligible students in person at the university campus via a QR code linking to the survey, achieving a response rate of 67%. Because all survey items were set as mandatory prior to final submission, there were no missing data in the completed responses. Participation was voluntary and anonymous. The study was conducted after approval by the institutional Research Ethics Committee, and informed consent was obtained electronically before participants accessed the questionnaire.

### Data Analysis

Data were analyzed using Jamovi software (version 2.3). Descriptive analyses were conducted to calculate means, standard deviations, and frequencies. Normality was examined through skewness and kurtosis coefficients and by visual inspection of histograms and Q-Q plots. Pearson bivariate correlations were calculated between the study-difficulties variable and the self-efficacy dimensions. Bonferroni correction was applied to reduce the probability of type I error; therefore, statistical significance was interpreted using an adjusted threshold of *p* < .003.

## Results

The study-difficulties variable was calculated as the mean score of items related to academic performance, content mastery, examination preparation, opportunities to clarify doubts, and time management. The mean score for perceived difficulties during study was 2.13±0.74. Self-efficacy dimensions presented relatively high mean scores: academic self-efficacy (8.08 ± 1.34), self-efficacy in training regulation (8.40 ± 1.26), proactive self-efficacy (8.09 ± 1.39), social-interaction self-efficacy (8.39 ± 1.33), and academic-management self-efficacy (8.59 ± 1.31).

The distributions of the analyzed variables were approximately normal. Skewness ranged from − 0.142 to − 0.879, and kurtosis ranged from − 0.417 to 0.804. Histograms showed approximately symmetric curves, and Q-Q plots indicated adequate alignment between observed and expected quantiles. For the academic-management self-efficacy variable, a cubic transformation was applied due to mild skewness and kurtosis; after transformation, the distribution approximated a Gaussian curve.

Pearson correlation analysis indicated statistically significant negative correlations between study difficulties and all self-efficacy dimensions at the Bonferroni-adjusted level (*p* < .003). The strongest association was observed between study difficulties and academic self-efficacy (*r* = − .530; IC 95% [-0.650; − 0.385]; R² = 0.281 ), followed by self-efficacy in training regulation (*r* = − .414; IC 95% [− 0.553; −0.252], R² = 0.171). Proactive self-efficacy (*r* = − .388; IC 95% [− 0.531; −0.223], R² = 0.151), social-interaction self-efficacy (*r* = − .373; IC 95% [− 0.518; −0.207], R² = 0.139), and academic-management self-efficacy (*r* = − .377; IC 95% [− 0.521; −0.211], R² = 0.142) also showed negative correlations of similar magnitude. Table 1 shows the correlations between study difficulties and self-efficacy.

**Table 1:** Correlation matrix between study difficulties and self-efficacy dimensions

Variable	1. Study difficulties	2. Academic self-efficacy	3. Self-efficacy in training regulation	4. Proactive self-efficacy	5. Social-interaction self-efficacy	6. Academic-management self-efficacy
1. Study difficulties	—					
2. Academic self-efficacy	− 0.530*	—				
3. Self-efficacy in training regulation	− 0.414*	0.806*	—			
4. Proactive self-efficacy	− 0.388*	0.804*	0.904*	—		
5. Social-interaction self-efficacy	− 0.373*	0.693*	0.840*	0.794*	—	
6. Academic-management self-efficacy	− 0.377*	0.767*	0.879*	0.785*	0.787*	—

Pearson correlations. *p < .003 after Bonferroni correction

These results support the hypothesis that higher perceived self-efficacy is associated with lower perceived study difficulties among early medical students. Students who reported stronger beliefs in their ability to learn, organize, persist, and manage academic demands tended to perceive lower difficulties related to performance, content mastery, examinations, time management, and access to academic support.

## Discussion

This study found that higher self-efficacy was associated with lower perceived study-related difficulties among first-cycle medical students. The negative correlations observed across all self-efficacy domains suggest that students who believe they can organize, regulate, and manage their academic activities tend to report fewer challenges related to performance, content mastery, examination preparation, time management, and access to learning support.

Academic self-efficacy showed the strongest association with study difficulties. This finding is theoretically consistent with Bandura’s social cognitive theory, which proposes that efficacy beliefs influence effort, persistence, and resilience in the face of obstacles [1, pp. 37–42]. Students who perceive themselves as capable of understanding and applying academic content may be more likely to engage actively with demanding topics, persist after poor performance, and seek strategies that improve comprehension rather than avoid difficult tasks. This stronger relationship compared to regulatory and management dimensions likely reflects the fact that academic self-efficacy represents a more proximal appraisal of learning ability and content comprehension, whereas self-regulation and management operate as instrumental resources for organizing the study routine.

Self-efficacy in training regulation also showed a meaningful negative correlation with perceived difficulties. This result supports the idea that academic success in medical education is not determined only by intellectual ability or content exposure, but also by students’ capacity to regulate their learning process. Self-regulated learners plan their study routines, monitor performance, adjust strategies, and evaluate outcomes [3, 8]. In the early semesters of medical school, these regulatory behaviors may be especially important because students are still adapting to new academic expectations and workloads.

The correlations involving proactive self-efficacy, social-interaction self-efficacy, and academic-management self-efficacy were slightly weaker but still significant. However, although these associations reached statistical significance, their practical and educational relevance warrants cautious interpretation given their weak-to-moderate magnitude. These dimensions may contribute indirectly to the reduction of academic difficulties. For example, proactive students may seek clarification, use available resources, and participate more actively in learning opportunities. Students with higher social-interaction self-efficacy may be more comfortable asking questions, working with peers, and communicating with faculty. Academic-management self-efficacy may support organization of curricular demands and institutional routines. Together, these findings suggest that self-efficacy is a multidimensional resource that supports both academic and interpersonal adaptation.

Taken together, these findings provide a more differentiated view of the relationship between self-efficacy and perceived study-related difficulties in early medical training. Although all self-efficacy domains were significantly associated with lower perceived difficulties, the associations were not of equal magnitude. Academic self-efficacy showed the strongest relationship, followed by self-efficacy in training regulation, whereas proactive, social-interaction, and academic-management self-efficacy showed comparatively smaller associations. This pattern suggests that efficacy beliefs more directly related to learning academic content and regulating the learning process may be particularly relevant to how students perceive and manage study-related challenges during the early stages of medical education. At the same time, the significant associations observed across all domains reinforce the multidimensional nature of self-efficacy and suggest that students’ academic experiences involve not only confidence in learning but also beliefs about their capacity to organize, seek support, interact, and navigate academic demands.

The findings have practical implications for medical education. First, student-support programs should not focus exclusively on remediation after poor academic performance. Instead, institutions may benefit from preventive interventions that strengthen self-efficacy and self-regulated learning during the first semesters. Workshops on evidence-based study strategies, structured mentoring, peer support, guided reflection, and formative feedback may help students transform perceived difficulties into opportunities for strategy adjustment [9, 10]. Second, faculty development initiatives can encourage teachers to provide constructive feedback, model effective learning strategies, and create classroom climates that normalize help-seeking and academic vulnerability.

The study also reinforces the need to connect academic support with mental health promotion. Perceived incapacity, repeated failure, and poor time management may increase stress and reduce engagement. Conversely, students who develop realistic beliefs in their capacity to learn and regulate their academic behavior may experience greater persistence and emotional stability. This is particularly relevant in medical education, where psychological distress and academic pressure are frequently reported.

This study has limitations. The sample was selected by convenience and was restricted to a single institution, which limits generalizability. The cross-sectional design prevents causal inference; therefore, it is not possible to determine whether higher self-efficacy reduces perceived difficulties or whether lower difficulties strengthen self-efficacy. In addition, the use of self-report instruments may be influenced by perception bias and social desirability. Future studies should use longitudinal designs, mixed methods, and intervention-based approaches to examine how self-efficacy changes across medical training and whether targeted psychoeducational programs can improve academic adaptation and well-being. Furthermore, very high intercorrelations among the self-efficacy subscales may point to limited discriminant validity, suggesting that these factors capture largely redundant aspects of the construct. To address this issue, future studies should employ multivariable modeling and structural equation modeling (SEM) to formally establish whether these dimensions represent distinct facets or a broad general factor, and examine their unique latent associations with perceived study-related difficulties.

## Conclusions

Higher perceived self-efficacy was associated with lower perceived study-related difficulties among first-cycle medical students. Academic self-efficacy and self-efficacy in training regulation showed the strongest associations, suggesting that confidence in one’s ability to learn and regulate the educational process may be particularly relevant during the transition into medical school. These findings support the development of institutional strategies that promote self-regulated learning, academic planning, autonomy, and early support for students facing perceived study-related difficulties. By strengthening self-efficacy, medical schools may contribute not only to academic performance but also to student persistence and well-being.

## Supplementary Information

Below is the link to the electronic supplementary material.

Supplementary Material 1 (DOCX 566 KB)

Supplementary Material 2 (DOCX 60.1 KB)

## Data Availability

The datasets generated and/or analyzed during the current study are available from the corresponding author upon reasonable request and in accordance with institutional ethical guidelines.

## References

[1] Bandura A. Self-efficacy: the exercise of control. Volume 3. New York: W. H. Freeman; 1997. pp. 37–42.

[2] Lopes JM, Castro JGF, Peixoto JM, Moura EP. Self-efficacy of medical students in two schools with different education methodologies (Problem-Based Learning versus Traditional). Rev Bras Educ Med. 2020;44(2):e047. 10.1590/1981-5271v44.2-20190187.ING.

[3] Zimmerman BJ Becoming a self-regulated learner: an overview. Theory Into Pract. 2002;41(2):64–70. 10.1207/s15430421tip4102_2.

[4] Abrams MP, Salzman J, Espina Rey A, Daly K Impact of providing peer support on medical students’ empathy, self-efficacy, and mental health stigma. Int J Environ Res Public Health. 2022;19(9):5135. 10.3390/ijerph19095135. PMC9099875 35564535

[5] Jones D, McCalla M, Beverly EA Measuring grit, self-efficacy, curiosity, and intolerance of uncertainty in first-generation college and first-generation osteopathic medical students. BMC Med Educ. 2023;23:190. 10.1186/s12909-023-04181-9. PMC10043857 36978030

[6] Zhang X, et al Regulatory emotional self-efficacy and psychological distress among medical students: multiple mediating roles of interpersonal adaptation and self-acceptance. BMC Med Educ. 2022;22:283. 10.1186/s12909-022-03338-2. PMC9011952 35421953

[7] Dunlosky J, Rawson KA, Marsh EJ, Nathan MJ, Willingham DT Improving students’ learning with effective learning techniques: promising directions from cognitive and educational psychology. Psychol Sci Public Interest. 2013;14(1):4–58. 10.1177/1529100612453266. 26173288

[8] Polydoro SAJ, Guerreiro-Casanova DC. Escala de auto-eficácia na formação superior: construção e estudo de validação. Aval Psicol. 2010;9(2):267–78.

[9] Franz A, Oberst S, Peters H, Berger R, Behrend R How do medical students learn conceptual knowledge? High-, moderate- and low-utility learning techniques and perceived learning difficulties. BMC Med Educ. 2022;22:250. 10.1186/s12909-022-03283-0. PMC8988315 35387637

[10] Ferreira MRC, Fernandes RAF, Malloy-Diniz LF, Ibiapina C Autoeficácia ou inteligência para predizer o sucesso acadêmico ou profissional? Rev Bras Educ Med. 2025;49(2):e057. 10.1590/1981-5271v49.1-2023-0010.

