# Supplementary material for: Self-Efficacy and Perceived Study-Related Difficulties in Early Medical Training: A Correlational Study
Source: Med Sci Educ. 2026 Sep 26;36(6):2993–8. doi: 10.1007/s40670-026-02895-1 (PMC13759867; doi:10.1007/s40670-026-02895-1)
Supplement: Supplementary file 1 [file 40670_2026_2895_MOESM1_ESM.docx]

Este instrumento foi desenvolvido para nos ajudar a identificar a auto-eficácia acadêmica de estudantes do ensino superior. Por favor, indique quanto você se percebe capaz de realizar as situações propostas em cada uma das questões que se seguem, considerando sua experiência de formação atual. Marque sua resposta em uma escala de (1) a (10), considerando um contínuo entre pouco e muito. Não existem respostas certas ou erradas. Suas respostas são confidenciais. Por favor, não deixe itens em branco.

Nº	Item	Pouco capaz<......> Muito capaz
1.	Quanto eu sou capaz de aprender os conteúdos que são necessários à minha formação?	1 2 3 4 5 6 7 8 9 10
2.	Quanto eu sou capaz de utilizar estratégias cognitivas (processo de adquirir um conhecimento) para facilitar minha aprendizagem?	1 2 3 4 5 6 7 8 9 10
3.	Quanto eu sou capaz de demonstrar, nos momentos de avaliação, o que aprendi durante meu curso?	1 2 3 4 5 6 7 8 9 10
4.	Quanto eu sou capaz de entender/compreender as exigências do meu curso?	1 2 3 4 5 6 7 8 9 10
5.	Quanto eu sou capaz de expressar minha opinião quando outro colega de sala discorda de mim?	1 2 3 4 5 6 7 8 9 10
6.	Quanto eu sou capaz de pedir ajuda, quando necessário, aos colegas nas atividades do curso?	1 2 3 4 5 6 7 8 9 10
7.	Quanto eu sou capaz de reivindicar atividades extracurriculares relevantes para a minha formação?	1 2 3 4 5 6 7 8 9 10
8.	Quanto eu sou capaz de planejar ações para atingir minhas metas profissionais?	1 2 3 4 5 6 7 8 9 10
9.	Quanto eu sou capaz de refletir sobre a realização de minhas metas de formação?	1 2 3 4 5 6 7 8 9 10
10.	Quanto eu sou capaz de selecionar, dentre os recursos oferecidos pela instituição, o mais apropriado à minha formação?	1 2 3 4 5 6 7 8 9 10
11.	Quanto eu sou capaz de aplicar o conhecimento aprendido no curso em situações práticas?	1 2 3 4 5 6 7 8 9 10
12.	Quanto eu sou capaz de estabelecer condições (de tempo/espaço/lugar) para o desenvolvimento/realização dos trabalhos solicitados pelo curso?	1 2 3 4 5 6 7 8 9 10
13.	Quanto eu sou capaz de trabalhar em grupo?	1 2 3 4 5 6 7 8 9 10
14.	Quanto eu sou capaz de compreender os conteúdos abordados no curso?	1 2 3 4 5 6 7 8 9 10

15.	Quanto eu sou capaz de manter-me atualizado sobre as novas tendências profissionais na minha área de formação?	1 2 3 4 5 6 7 8 9 10
16.	Quanto eu sou capaz de tomar decisões relacionadas à minha formação?	1 2 3 4 5 6 7 8 9 10
17.	Quanto eu sou capaz de cooperar com os colegas nas atividades do curso?	1 2 3 4 5 6 7 8 9 10
18.	Quanto eu sou capaz de esforçar-me nas atividades acadêmicas?	1 2 3 4 5 6 7 8 9 10
19.	Quanto eu sou capaz de definir, com segurança, o que pretendo seguir dentre as diversas possibilidades de atuação profissional que existem na minha área de formação?	1 2 3 4 5 6 7 8 9 10
20.	Quanto eu sou capaz de procurar auxílio dos professores para o desenvolvimento de atividades do curso?	1 2 3 4 5 6 7 8 9 10
21.	Quanto eu sou capaz de motivar-me para fazer as atividades ligadas ao curso?	1 2 3 4 5 6 7 8 9 10
22.	Quanto eu sou capaz de estabelecer minhas metas profissionais?	1 2 3 4 5 6 7 8 9 10
23.	Quanto eu sou capaz de estabelecer bom relacionamento com meus professores?	1 2 3 4 5 6 7 8 9 10
24.	Quanto eu sou capaz de cumprir o desempenho exigido para aprovação no curso?	1 2 3 4 5 6 7 8 9 10
25.	Quanto eu sou capaz de contribuir com ideias para a melhoria do meu curso?	1 2 3 4 5 6 7 8 9 10
26.	Quanto eu sou capaz de terminar trabalhos do curso dentro do prazo estabelecido?	1 2 3 4 5 6 7 8 9 10
27.	Quanto eu sou capaz de planejar a realização das atividades solicitadas pelo curso?	1 2 3 4 5 6 7 8 9 10
28.	Quanto eu sou capaz de perguntar quando tenho dúvida?	1 2 3 4 5 6 7 8 9 10
29.	Quanto eu sou capaz de estabelecer amizades com os colegas do curso?	1 2 3 4 5 6 7 8 9 10
30.	Quanto eu sou capaz de atualizar os conhecimentos adquiridos no curso?	1 2 3 4 5 6 7 8 9 10
31.	Quanto eu sou capaz de resolver problemas inesperados relacionados à minha formação?	1 2 3 4 5 6 7 8 9 10
32.	Quanto eu sou capaz de preparar-me para as avaliações?	1 2 3 4 5 6 7 8 9 10
33.	Quanto eu sou capaz de aproveitar as oportunidades de participar em atividades extracurriculares?	1 2 3 4 5 6 7 8 9 10
34.	Quanto eu sou capaz de buscar informações sobre os recursos ou programas oferecidos pela minha instituição?	1 2 3 4 5 6 7 8 9 10

Fonte: (POLYDORO;GUERREIRO, 2010)
