# Supplementary material for: Self-Efficacy and Perceived Study-Related Difficulties in Early Medical Training: A Correlational Study
Source: Med Sci Educ. 2026 Sep 26;36(6):2993–8. doi: 10.1007/s40670-026-02895-1 (PMC13759867; doi:10.1007/s40670-026-02895-1)
Supplement: Supplementary file 2 [file 40670_2026_2895_MOESM2_ESM.docx]

DIFICULDADES DURANTE OS ESTUDOS		2	3	4	5
Atingir um bom desempenho					
Dominar a quantidade de conteúdo aprendido					
Preparar para as provas de forma eficiente					
Falta de oportunidade de tirar as dúvidas					
Otimizar o tempo de estudo/pressão do tempo					

(1=dificuldades extremas; 2= grandes dificuldades, 3= dificuldades moderadas , 4= pequenas dificuldades e 5=sem dificuldades)
